# Real-Time Dynamic Observation of Micro-Friction on the Contact Interface of Friction Lining

**DOI:** 10.3390/ma11030369

**Published:** 2018-03-02

**Authors:** Cunao Feng, Dekun Zhang, Kai Chen, Yongbo Guo

**Affiliations:** 1 School of Mechatronic Engineering, China University of Mining and Technology, Xuzhou 221116, China; cumtfca@126.com (C.F.); guoyongbo5@126.com (Y.G.); 2 School of Materials Science and Engineering, China University of Mining and Technology, Xuzhou 221116, China; cumtck@cumt.edu.cn

**Keywords:** friction lining, in situ microscopic observation, adhesive friction, hysteresis friction

## Abstract

This paper aims to investigate the microscopic friction mechanism based on in situ microscopic observation in order to record the deformation and contact situation of friction lining during the frictional process. The results show that friction coefficient increased with the shear deformation and energy loss of the surfacee, respectively. Furthermore, the friction mechanism mainly included adhesive friction in the high-pressure and high-speed conditions, whereas hysteresis friction was in the low-pressure and low-speed conditions. The mixed-friction mechanism was in the period when the working conditions varied from high pressure and speed to low pressure and speed.

## 1. Introduction

**Figure 1:**
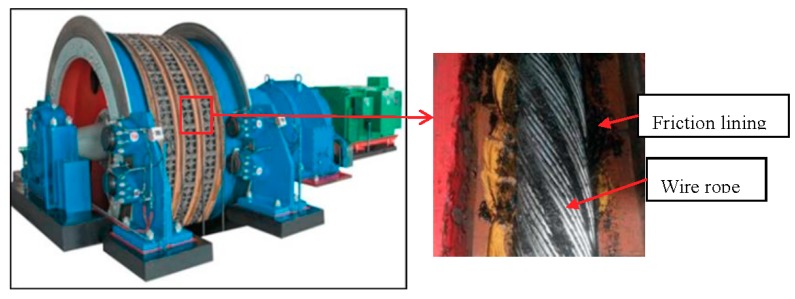
The schematic representation of the contact between the rope and lining.

The friction hoist system delivers coal, and lifts and lowers materials and staff. It mainly depends on friction lining to bear load and provides a friction force between the wire rope and lining. The schematic representation of the contact between the rope and lining is shown in Figure 1. The properties of friction lining directly influence the hoisting capacity, the working efficiency and the safety reliability of the friction hoist [1].

At present, studies of friction lining are mainly based on black-box theoretical derivation and the analysis of testing results. Ye et al. [2] analyzed the wear surface topography of the wire rope and friction lining at the micro level using a scanning electron microscope and revealed that the wear mechanism mainly included abrasive wear, fatigue wear and adhesive wear. Peng et al. [3,4,5] studied the thermal stress coupled field, heat conduction and heat dissipation of lining in the high-speed sliding condition and established a thermal stress theoretical model based on spiral contact characteristics; eventually, they proved experimentally that the thermal stress concentration occurred in the high-temperature contact area. The influences of tension at both ends of the wire rope on the friction coefficient were explored theoretically and experimentally. Pinnington [6,7] built a hysteresis sliding model and discussed the relationships between the adhesive friction coefficient and the sliding friction coefficients after studying the friction coefficient of macromolecule polymer rubber in the smooth and rough surface. 

When inventing visual testing machines, research on the friction interface by real-time observation based on the white box test has been rapidly developed [8,9]. Zhang et al. [10] observed in situ contact friction interfacial behaviors of friction lining and high-strength transparent toughened glass using a high-speed camera; that way, the real contact area of the interface could be calculated using the gray-level method to reveal the wear mechanism of friction lining. Chromik et al. [11] studied sliding conditions, friction wear behaviors and the structural chemical changes of sub-surfaces in the contact interface of the cladding layer and sapphire that were observed using optical microscopy and Raman spectroscopy. Loeve et al. [12] studied the static friction of stainless wire rope and rubber in the relative sliding initiation stage with video recorders, thus the main factors affecting static friction were illustrated. Brandon et al. [8] designed an experimental machine to observe in situ the real contact area of acrylonitrile-butadiene rubber and transparent glass in various conditions and proved that contact area had an obvious hysteresis effect in the loading and unloading process. Gong [13] studied the factors influencing frequency and amplitude of bond-slip action based on kinetic model of interfacial friction systems and characteristics of the bond-slip action tested by atomic force microscopy. Horst [14] summarized the interfacial friction phenomena at the macro, micro and nano level, and observed the real-time producing process of abrasive particles using SEM. Teng [15] observed in situ interfacial torsional friction behaviors of PMMA and 45# steel and distinguished an adhesive region and slip region of the friction interface. Bhushan [16] proved that the real contact of the friction surface only occurred in the micro bulges, which made real contact area much smaller than the nominal contact area. However, research on the sliding process of friction lining and wire rope, based on the white box test, is still scarce.

In this paper, friction lining was microscopically observed in situ in the dynamic contact and friction process, and micro topographies of the real-time contact surface were recorded. Then the deformation tracks of the lining sub-surface were traced, and shear deformation of the sub-surface and enclosed area of the deformation tracks were calculated. Eventually, the relationship between friction coefficient and dynamic deformation of lining was established to reveal the micro friction mechanism of friction lining. 

## 2. Experimental Materials and Methods

K25 lining, as a research subject, is widely applied in friction hoists in China. It is generally made of high-strength heat-resistant resin, carrier resin and a variety of additives. At present, the matrix of friction lining in China is mainly phenolic resin modified by NBR. The fillers are montmorillonite, SiO_2_ and other space fillers. Meanwhile, the functional additives are rubber additives, including cross-linkers, a plasticizer, an accelerator, and an antioxidant agent [10,17]. The performance parameters are shown in Table 1.

**Figure 2:**
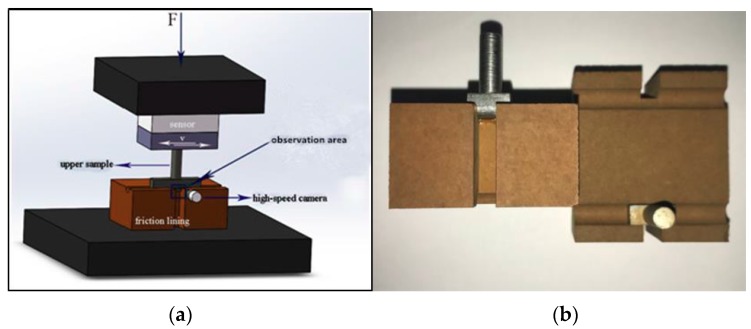
Experimental schematic figure of friction ling and circular column. (**a**) Experimental schematic diagram; (**b**) samples.

The test platform was a combination of the UMT-II friction tester (CETR) with a high-speed microscope (VW 9000, KEYENCE, Osaka, Japan) as shown in Figure 2. The size of the lining is 45 × 25 × 25 mm^3^ and the arc gutter with a radius of 3 mm is machined in the lining surface to simulate the actual contact conditions as shown in Figure 2. To simplify the experiment and easily observe it, a 316L stainless steel circular column is chosen to simulate the wire rope. Furthermore, a gutter with a depth of 7.8 mm and a width of 6 mm is machined to observe the dynamic contact interface of the lining and circular column. 

During the experiment, the contact load is applied on the friction lining through the 316L stainless steel circular column. Then reciprocating friction experiments begin at various speeds after contact time of 5 s and sliding distance of 4 mm. The lens of the high-speed microscope camera (VW 9000) is aligned with the contact interface and used to observe real-time contact and sliding of friction lining and stainless steel. The parameters of the reciprocating frictional sliding experiment are shown in Table 2. The camera frame rate is 9000 fps and the resolution of each pixel is 640 × 480. The horizontal and vertical axes are chosen as reference axes. In addition, marked points *s* and *l* in the stainless steel and friction lining are marked. The tracks of the marked points *s* and *l* relating to axes are recorded, respectively. The deformation is measured by the marked points in the video, frame by frame, which is the change in position between the two frames of the marked points. According to the tracks of the marked points *s* and *l*, the horizontal shear deformation ∆X and ∆Y relating to X and Y axis in stroke and return stage are obtained. The high-speed microscope camera’s accuracy of measuring the distance can reach 0.01 μm. The enclosed area of the deformation tracks per cycle in reciprocating the sliding process are calculated.

## 3. Results and Discussion

### 3.1. Micro Dynamic Observation of the Contact Interface in a Single Friction Period

**Figure 3:**
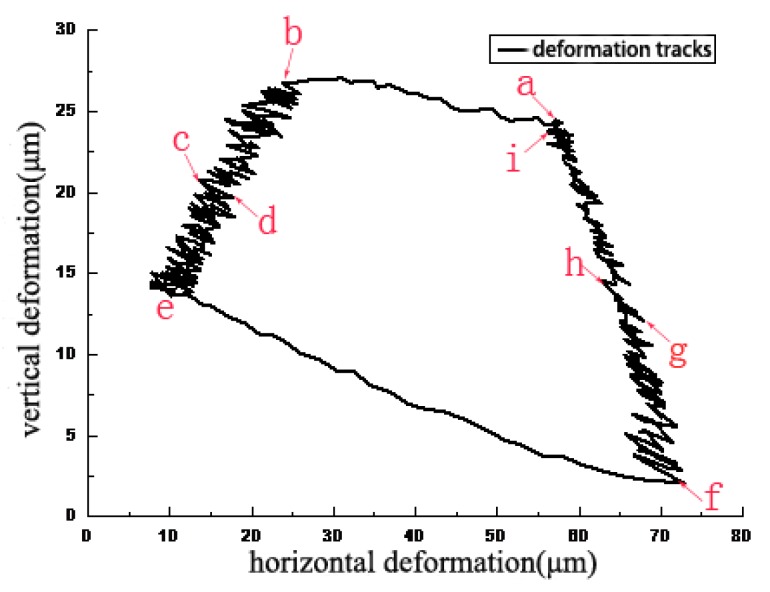
Deformation track of lining surface marked point *l* in a single frictional period (P = 2 MPa, V = 3 mm/s).

**Figure 4:**
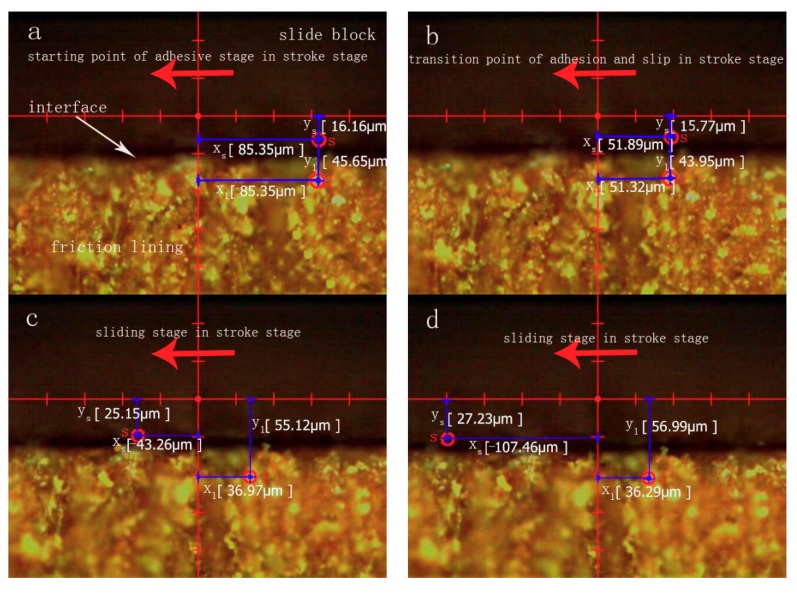

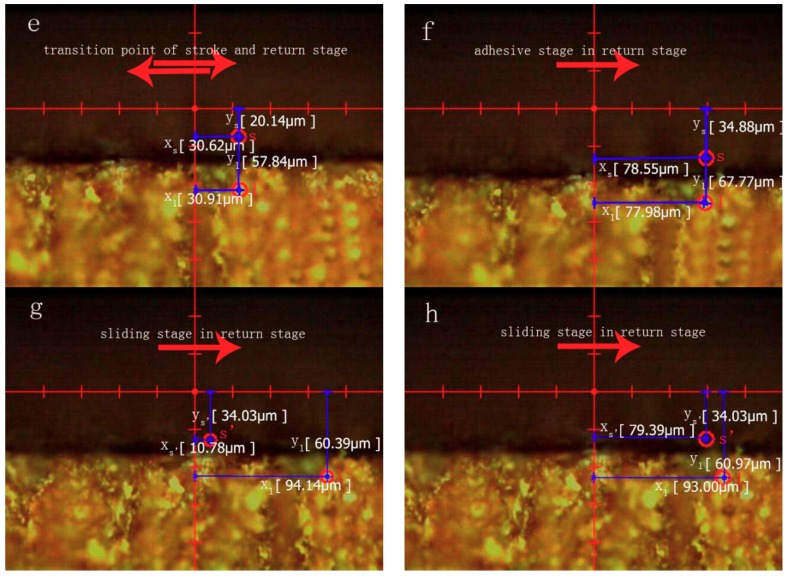
Interfacial contact situations of various stages in a single frictional period (P = 2 MPa, V = 3 mm/s). Adhesive stage in stroke stage: (**a**) 0 s, (**b**) 0.18 s; Sliding stage in stroke stage: (**c**) 0.58 s, (**d**) 1.05 s; Adhesive stage in return stage: (**e**) 1.24 s, (**f**) 1.3 s; Sliding stage in return stage (**g**) 1.76 s, (**h**) 2.2 s.

Figure 3 illustrates the deformation track of the marked point *l* in a single friction cycle relating to the X and Y axes when contact pressure and sliding speed are 2 MPa and 3 mm/s, respectively. The points *a–i* are chosen around the counter clockwise rotation of the deformation track. Then the instantaneous contact situation of the contact interface and the position change of marked points *s* and *l* in the moments of points *a–i* are shown in Figure 4. The first half of the sliding cycle, that is, the process of sliding from the initial zero position to the set position, is defined as the stroke stage. The latter half of the cycle is defined as return stage, which is the process of sliding from the set position to the initial zero position at the same speed. The deformation track of marked point *l* towards left from point *a* to point *b* in the stroke stage is shown in Figure 3. Figure 4a,b shows that both horizontal positions X_s_ and X_l_ of marked points *s* and *l* deform from 85.35 μm to 51.89 μm. This indicates that no relative displacement occurred between marked points *s* and *l* in this period, which is defined as adhesive stage.

The deformation track of marked point *l* downwards and left from point *b* to point *e* in the stroke stage is shown in Figure 3, where an instantaneous deformation track (from point *c* to point *d*) is chosen. Figure 4c,d shows that the position X_l_ of marked point *l* holds at about 36.97 μm, the position X_s_ of marked point *s* slides from −43.26 μm to −107.46 μm, and the position Y_l_ of marked point *l* deforms from 55.12 μm to 56.99 μm, the position Y_s_ of marked point *s* moves from 25.15 μm to 27.23 μm. A position change means that the vertical distance ∆Y between marked points *l* and *s* holds at about 29.97 μm and an instantaneous horizontal displacement ∆X between marked points *l* and *s* occurs in the period which can be defined as the sliding stage. Moreover, the interfacial contact from point *c* to point *d* can represent the typical contact situation of the period from point *b* to point *e*, therefore the total period from point *b* to point *e* can be defined as a sliding stage.

The total return stage can also be divided into two stages including adhesive stage and sliding stage. A position change of marked point *l* relating to that of marked point *s* in the stroke stage is also same as in the return stage. The deformation track of marked point *l* downwards and right from point *e* to point *f* in the return stage is shown in Figure 3. Figure 4e,f shows that X_l_ and X_s_ deform from 30.62 μm to 97.54 μm due to interfacial adhesive force. This phenomenon means that no relative displacement occurs in the period which can be defined as the adhesive stage.

The deformation track of marked point *l* upwards and left from point *f* to point *i* in the return stage is shown in Figure 3, where a typical instantaneous deformation track (from point *g* to point *h*) is chosen. A new marked point *s’* is chosen because the marked point *s* in the circular column moves out the field of view. Figure 4g,h shows that X_s_^,^ rapidly slides from 10.78 μm to 79.39 μm, where X_l_, Y_l_ and Y_s’_ hold at about 94.14 μm, 60.39 μm, and 34.03 μm, respectively. The phenomenon reveals that ∆Y remains static and ∆X rapidly increases in the period, which can be defined as the sliding stage.

### 3.2. Microscopic Friction Mechanism of Lining Contact Interface in a Single Friction Period

**Figure 5:**
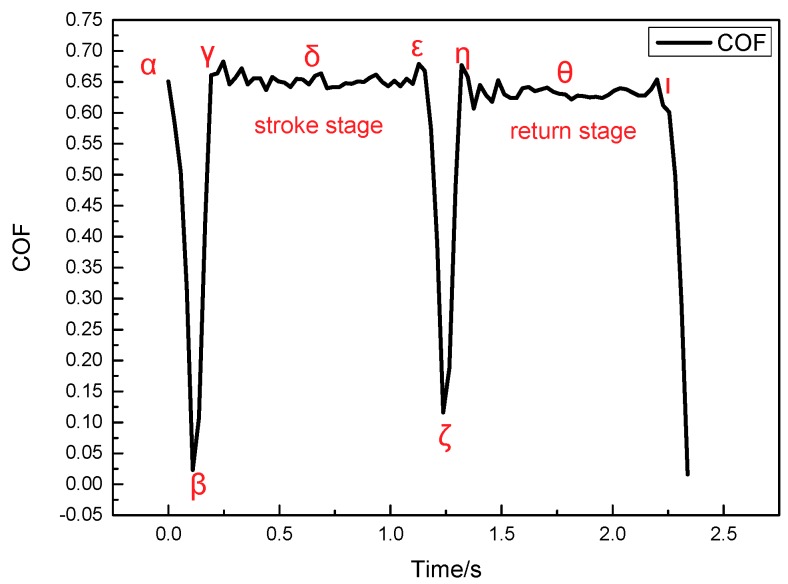
Law curve of friction coefficient in a single friction period (P = 2 MPa, V = 3 mm/s).

**Figure 6:**
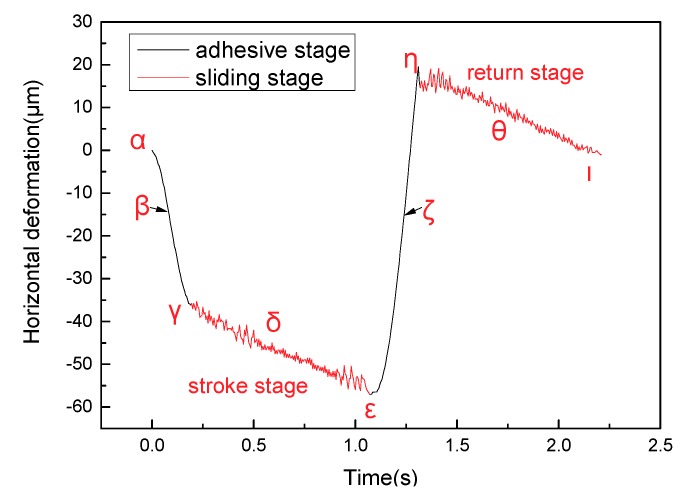
Shear deformation curve of lining surface marked point *l* in a single friction period (P = 2 MPa, V = 3 mm/s).

**Figure 7:**
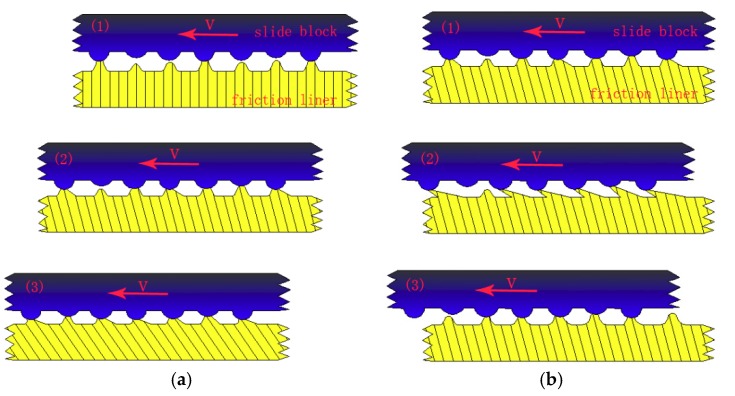
Diagrams of adhesive mechanism in various stages of the stroke stage. (**a**) adhesive stage; (**b**) sliding stage.

Figure 5 shows the curve of friction coefficient in a single friction period at the contact pressure of 2 MPa and the sliding speed of 3 mm/s. Figure 6 shows the shear deformation curve of lining surface in the sliding frictional process. Seven *α*–*θ* marking points in the curve are obtained due to the law of friction coefficient and shear deformation. Friction coefficient from point *α* to point *β* rapidly decreases in the stroke stage (shown in Figure 5). The interfacial contact situation of this period (adhesive stage in the stroke stage in Figure 4) shows that this stage is in a state of adhesion. Then point *β* in Figure 6 indicates that the residual shear deformation of the lining surface is elastically recovered, which leads the sticky points on the contact surface to be in a state of equilibrium, and for adhesive force to decrease to zero. After point *β*, the friction coefficient of the lining rapidly increases and reaches a maximum in point *γ*. The reason is that the contact interface is still in a state of adhesion and no relative displacement occurs. Furthermore, with the development of slip, the adhesive shear deformation of the lining surface rapidly increases, and sticky points deform, contributing to the occurrence of new sticky points between the smaller micro bulge and the surface of the circular column. The phenomenon can lead to the increase in sticky points and real contact area. Thus, adhesive friction coefficient increases (adhesive friction mechanism in Figure 7: stage 1 is a state of adhesion, stage 2 and stage 3 are the adhesive deformation stage and no relative displacement). Then ∆X_l_ reaches a maximum (34.03 μm) as the adhesive friction coefficient reaches a maximum. Overall, the variety of friction coefficient from point *α* to point *γ* is caused by adhesive friction, and a positive proportional relationship occurs between ∆X_l_ in the adhesive stage and the adhesive friction coefficient.

Figure 5 shows the friction coefficient from point *γ* to point *ε* in the stroke stage presenting a state of steady situation. The interfacial contact situation (sliding stage in the stroke stage in Figure 4) shows that the stage is in a state of sliding situation. Moreover, the occurrence of an instantaneous displacement contributes to the break of the sticky points, and new sticky points are built in new places (the adhesive mechanism in the sliding stage in Figure 7: stage 1 is a state of adhesion, stage 2 is the adhesive deformation stage, stage 3 has an instantaneous displacement and new sticky points are built in new places). A dynamic equilibrium is built between the formation and break of sticky points in the reciprocating frictional process. The repeated tensile, fracture and relaxation of molecular bonds occurs in the process of the formation of new bonds and the fracture of old bonds [18]. This contributes to the relatively steady variety of ∆X_l_ and real contact area. Adhesive friction, therefore, is in a state of steady situation. Furthermore, the frictional mechanism in adhesive and sliding stages reveals that parts of the sticky points are in a state of fracture in the sliding stage [18], causing the adhesive friction coefficient in this period to be evidently smaller than that at the end of adhesive stage. Moreover, as the friction lining is a kind of viscoelastic material, tiny micro bulges in the contact surface generate repeated cyclic deformation when the surface of the friction lining is in a state of sliding situation in this period. Then, the repeated cyclic deformation can generate horizontal hysteresis force, hindering the movement of the circular column, and the behavior is defined as hysteresis friction [18]. Overall, the friction in this period includes adhesive and hysteresis friction.

Point *ε* is the transition point of the stroke and return stage (shown in Figure 5), surface shear deformation of the lining rapidly recovers after point *ε* (shown in Figure 6), and the friction coefficient rapidly decreases to a minimum at point *ζ*. The period from point *ζ* to point *η* is referred to as the adhesive stage, where real contact area gradually increases. Furthermore, the adhesive friction coefficient increases to a maximum at maximum shear deformation (66.63 μm) at the point *η*. The period from point *η* to point *θ* is called the sliding stage, where the real contact area and instantaneous sliding speed reach a steady situation. These behaviors contribute to a steady variation trend of friction coefficient. Overall, the variation trend in the return stage is the same as that in the stroke stage.

### 3.3. Friction Mechanism in the Total Experimental Process

**Figure 8:**
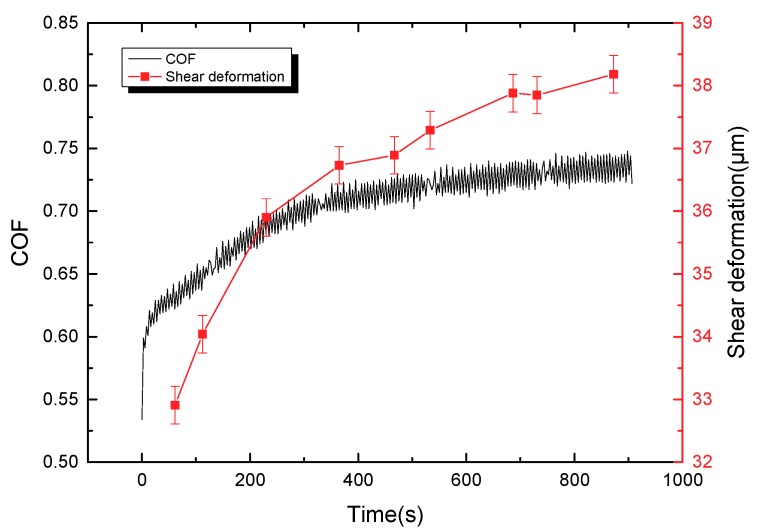
The surface shear deformation and friction coefficient of friction lining with time (P = 2 MPa, V = 3 mm/s).

Figure 8 shows that the friction coefficient and shear deformation of lining material with time in the sliding stage of the stroke stage; and the curve of the friction coefficient is similar to that of the shear deformation. The shear deformation of the lining surface increases in the sliding process, which causes the separation between polymer chains and fillers. The entanglement among polymer chains was broken, and aggregates are decomposed. The phenomena can damage the network structure of friction lining and contribute to the rapid decrease of storage modulus [19,20]. Moreover, the destroyed internal network structure can improve the movement of fillers and polymer chains, which can increase the internal friction force and release much more energy [19,21], leading to the increase of the loss modulus. Furthermore, based on Moore’s adhesion friction theory, the viscoelastic property of lining can be divided into adhesive and hysteresis friction coefficients. For hysteresis friction coefficient, the following Equation (1) is used:

(1) $$\mu_{h}=K_{h}\frac{E^{\prime \prime }}{{\left(E^{\prime }\right)}^{2}}p$$

where: *μ*_h_ is the hysteresis friction coefficient, *K*_h_ is a constant, *p* is the contact positive pressure, *E″* is the loss modulus and *E’* is the storage modulus.

Following Equation (2) was used for calculating adhesive friction coefficient:

(2) $$\mu_{a}=K_{a}\frac{1}{p^{r}}E^{\prime \prime }$$

where: *μ*_a_ is the adhesive friction coefficient, *K*_a_ is a constant and Exponent *r* = 0.2.

With the increase of surface shear deformation of the friction lining, the storage modulus decreases, and the loss modulus increases. The hysteresis and adhesive friction coefficients, therefore, increase with the viscoelastic property of the friction lining. Overall, the friction coefficient of friction lining increases with the shear deformation in the reciprocating frictional process.

**Figure 9:**
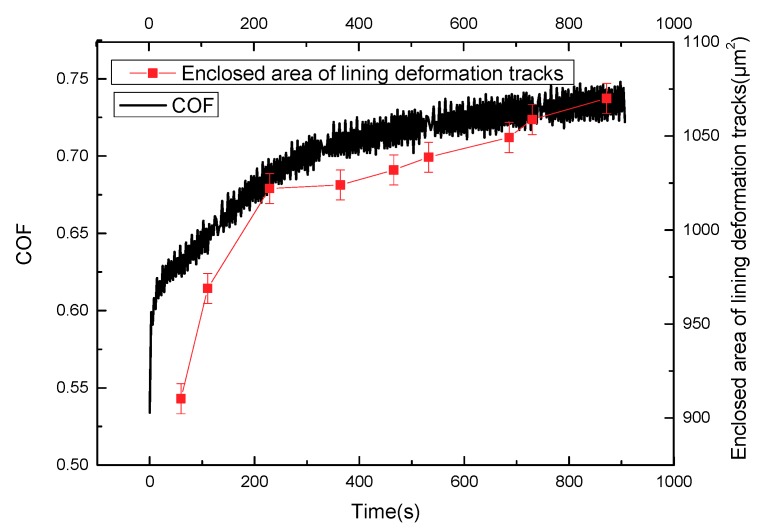
The enclosed area of surface deformation tracks and friction coefficient of friction lining with time (P = 2 MPa, V = 3 mm/s).

Figure 9 shows that the friction coefficient and enclosed area of the deformation tracks vary with time in the sliding stage of the stroke stage; and the curve of the friction coefficient is similar to that of the enclosed area of the deformation tracks. Moreover, the repeated cyclic deformation of the lining surface occurs in the sliding process, which leads to the relative movement among fillers and polymer chains. The movements can generate internal friction force in the network structure, hindering the deformation of the lining surface and, furthermore, generating horizontal force (hysteresis friction force) on the micro bulges of the lining contact interface to hinder the slip of circular column. The work of deformation in the process of deformation is greater than that released in the process of recovery. A part of the energy stored in the lining material is converted into hysteresis energy. The value of energy loss can be marked by the enclosed area of marked point *l* of the deformation tracks in a single period (shown in Figure 4).

With the increase of the enclosed area of deformation tracks on the lining surface, the loss modulus increases in the sliding process, leading to the increase of the internal friction force of material. This phenomenon can cause the hysteresis friction force of micro bulges on the contact interface to hinder the movement of slide. Overall, the friction coefficient of lining material increases with the increasing loss modulus in the reciprocating sliding process.

### 3.4. Influence from Sliding Speed on the Friction Mechanism of Friction Lining

**Figure 10:**
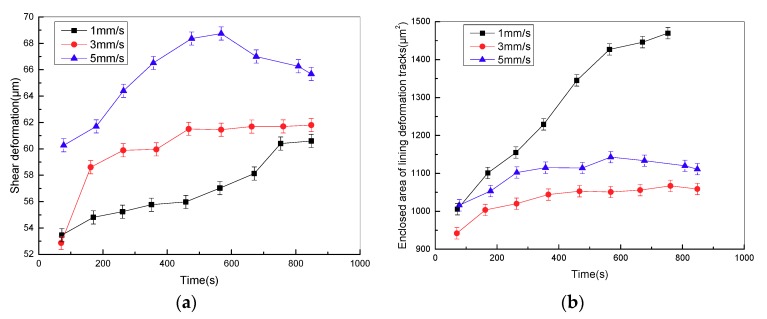

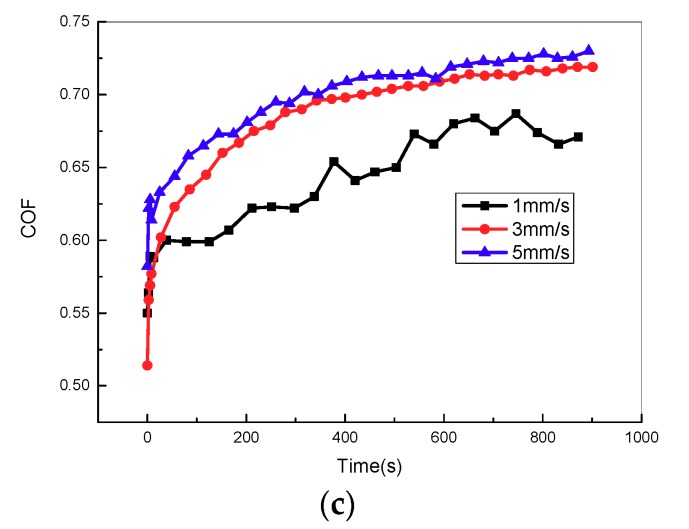
Variation curve of (**a**) surface shear deformation, (**b**) enclosed area of surface deformation tracks and (**c**) friction coefficient of friction lining with time under different sliding speeds.

Figure 10a shows the variation curve of the surface shear deformation of friction lining in the stroke stage with various sliding speeds at the contact pressure of 2 MPa. Surface shear deformation of friction lining increases with the increase of sliding speeds. Then the storage modulus decreases, and the loss modulus increases as the amount of surface shear deformation increases. Furthermore, with the increase of sliding speed, the frequency of cyclic stress applied on the surface of the friction lining increases. In the low-frequency zone, the moving distance of fillers and polymer chains in the network structure increases at the same time as the frequency increases, which means that the loss energy overcoming internal friction resistance increases, and the loss modulus of lining material increases [19]. The adhesive friction coefficient increases with the loss modulus based on Equation (2), as both frequency and strain can lead to the increase in the loss modulus of friction lining. In addition, the enclosed area of the lining surface deformation tracks firstly decreases, and then increases with the sliding speeds (shown in Figure 10b). This ultimately contributes to the hysteresis energy loss of friction lining presenting a trend that first decreases and then increases, and hysteresis friction presents the same variety trend. Consequently, the friction mechanism of friction lining mainly includes adhesive friction in the low-speed zone; and the friction mechanism of friction lining is adhesive and hysteresis frictions occur in the high-speed zone. 

Figure 10c shows the variation curve of the friction coefficient in the stroke stage with different sliding speeds at the contact pressure of 2 MPa. The friction coefficient increases with sliding speeds. In the low sliding speed zone, adhesive friction coefficient increases with the loss modulus based on Equation (2), as both frequency and strain can lead to the increase in the loss modulus of friction lining. In the high sliding speed zone, the storage modulus of friction lining relies more on strain than frequency under constant pressure, thus the increasing strain leads to the increase in hysteresis friction coefficient. Both frequency and strain can lead to the increase of the loss modulus, which contributes to the increase in adhesive friction coefficient. Therefore, the friction coefficient presents an increasing trend with the sliding speeds.

### 3.5. Influence from Contact Pressure on the Frictional Mechanism of Friction Lining

**Figure 11:**
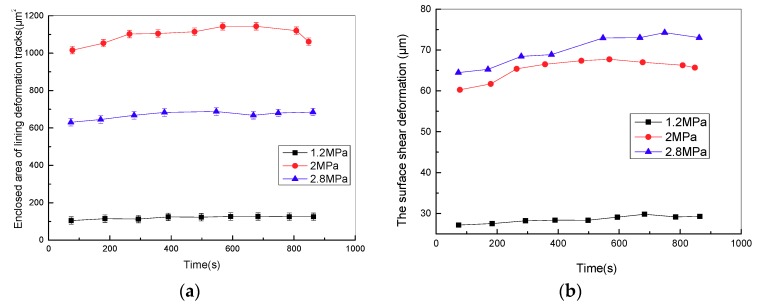

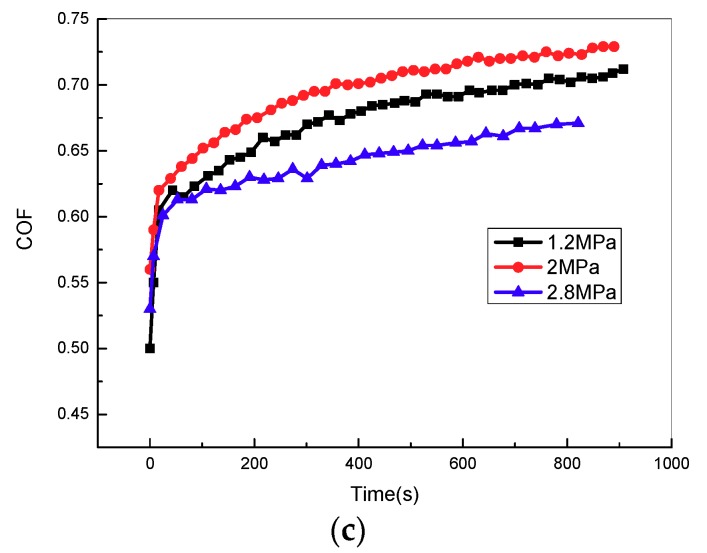
Variation curve of (**a**) enclosed area of surface deformation tracks, (**b**) surface shear deformation and (**c**) friction coefficient with time under different pressures.

Figure 11 shows the variation curve of the enclosed area of surface deformation tracks in the sliding stage of the stroke period with various pressures at the sliding speed of 5 mm/s. It is evident that the enclosed area of surface deformation tracks firstly increases and then decreases, which means that the hysteresis energy loss of friction lining increases first, and then decreases with pressure, and the hysteresis friction coefficient presents a similar trend to the hysteresis energy loss. Moreover, the surface shear deformation of lining in the stroke stage increases with contact pressure (shown in Figure 11b). This phenomenon results in the decrease of the storage modulus and the increase of the loss modulus of friction lining. Therefore, Equation (2) reveals that adhesive friction coefficient increases with the viscoelastic property of friction lining. Overall, in the low-pressure zone (1.2–2 MPa), the friction mechanism of friction lining mainly includes hysteresis friction, whereas in the high-pressure zone (2–2.8 MPa), the friction mechanism of friction lining mainly includes adhesive friction.

Figure 11c illustrates the variation curve of the friction coefficient in the stroke stage with various pressures at the sliding speed of 5 mm/s. The friction coefficient first increases, and then decreases with the increase of contact pressure. Hysteresis friction is the main friction mechanism of friction lining in the low-pressure zone. Equation (1) shows that the hysteresis friction coefficient of friction lining increases with contact pressure, leading to the increase of the friction coefficient. In the high-pressure zone, the main friction mechanism of friction lining is adhesive friction. Equation (2) shows that the adhesive friction coefficient of friction lining decreases with the increase of contact pressure, leading to the decrease of the friction coefficient. Overall, the friction coefficient increases first and then decreases with the increase of the contact pressure.

### 3.6. Friction Mechanism of Friction Lining under Various Conditions

**Figure 12:**
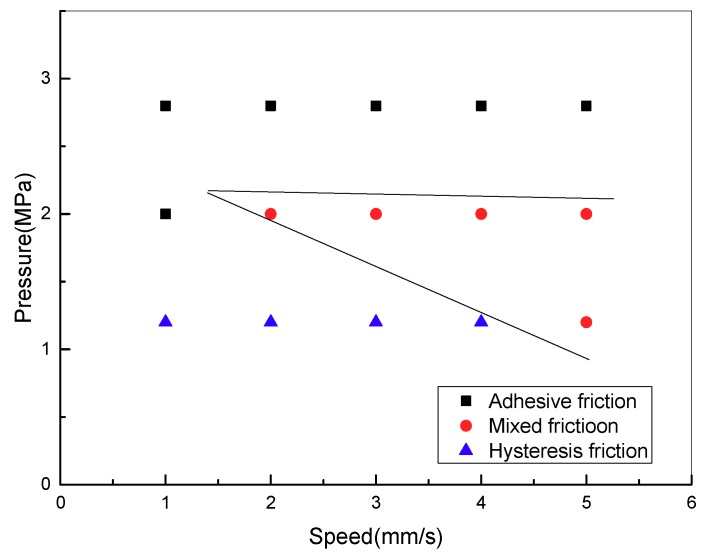
Friction mechanism map of friction lining under different conditions.

Based on the study of the influence of contact pressure and sliding speed on the lining friction mechanism, the friction mechanism of friction lining is achieved under the conditions of constant pressures and sliding speeds. Figure 12 shows the friction mechanism of the lining with contact pressure and sliding speed. Furthermore, three zones of friction mechanism are ensured based on the variety law. The friction mechanism of the friction lining mainly includes adhesive friction under high-pressure and high-speed conditions, whereas it mainly includes hysteresis friction under low-pressure and low-speed conditions. Then the friction mechanism includes adhesive and hysteresis friction as the working conditions change from high speed and pressure to low speed and pressure. 

## 4. Conclusions

Evident adhesive and sliding situations occur in the interface during the stroke and return stage. In the adhesive stage, no relative displacement occurs between friction pairs, surface deformation rate is high and real contact area increases. These result in the increase in interfacial adhesive force. Moreover, in the sliding stage, relative displacement occurs between friction pairs. Surface deformation rate is low and real contact area reaches a steady situation. These phenomena result in a steady situation of interfacial adhesive force. Furthermore, the main frictional mechanism in the adhesive stage is adhesive friction, and the frictional mechanism in the sliding stage includes adhesive and hysteresis frictions.

Adhesive and hysteresis frictions contribute to the friction coefficient of friction lining with an exponential increase with time. Moreover, the friction coefficient increases with the increase of sliding speeds. Furthermore, the frictional mechanism of friction lining mainly includes adhesive friction under high-pressure and high-speed conditions, whereas it mainly includes hysteresis friction under low-pressure and low-speed conditions. Then, the friction mechanism includes adhesive and hysteresis friction as the working conditions change from high speed and pressure to low speed and pressure.

## Figures and Tables

**Table 1:** Performance parameters of K25 friction lining.

Parameter	Value	Parameter	Value
Allowable friction coefficient (oil)	μ ≥ 0.25	Elongation	30%
Allowable surface pressure/MPa	<5	Shore hardness D	52–56
Tension/N/cm^2^	560	Density/g/cm^3^	1.4
Impact strength/KJ/m^2^	9.72	Tensile strength/MPa	19.10
Bending strength/MPa	26.22	Flexural modulus/MPa	920
Elongation at break%	10.6	Compressive strength/MPa	45.6

**Table 2:** Parameters of reciprocating frictional sliding experiment.

Parameter	Value
Pressure (MPa)	1.2, 2, 2.8
Sliding speed (mm/s)	1, 3, 5
Sliding distance (mm)	4
Experimental temperature (°C)	25
Experiment time (min)	15
